# AI-based prediction for the risk of coronary heart disease among patients with type 2 diabetes mellitus

**DOI:** 10.1038/s41598-020-71321-2

**Published:** 2020-09-02

**Authors:** Rui Fan, Ning Zhang, Longyan Yang, Jing Ke, Dong Zhao, Qinghua Cui

**Affiliations:** 1 Department of Biomedical Informatics, Department of Physiology and Pathophysiology, Center for Noncoding RNA Medicine, MOE Key Lab of Cardiovascular Sciences, School of Basic Medical Sciences, Peking University, 38 Xueyuan Rd, Beijing, 100191 China; 2 Beijing Key Laboratory of Diabetes Research and Care, Center for Endocrine Metabolism and Immune Diseases, Lu He Hospital Capital Medical University, Beijing, 101149 China

**Keywords:** Cardiology, Diseases, Medical research, Risk factors, Diagnosis

## Abstract

Type 2 diabetes mellitus (T2DM) is one common chronic disease caused by insulin secretion disorder that often leads to severe outcomes and even death due to complications, among which coronary heart disease (CHD) represents the most common and severe one. Given a huge number of T2DM patients, it is thus increasingly important to identify the ones with high risks of CHD complication but the quantitative method is still not available. Here, we first curated a dataset of 1,273 T2DM patients including 304 and 969 ones with or without CHD, respectively. We then trained an artificial intelligence (AI) model using randomly selected 4/5 of the dataset and use the rest data to validate the performance of the model. The result showed that the model achieved an AUC of 0.77 (fivefold cross-validation) on the training dataset and 0.80 on the testing dataset. To further confirm the performance of the presented model, we recruited 1,253 new T2DM patients as totally independent testing dataset including 200 and 1,053 ones with or without CHD. And the model achieved an AUC of 0.71. In addition, we implemented a model to quantitatively evaluate the risk contribution of each feature, which is thus able to present personalized guidance for specific individuals. Finally, an online web server for the model was built. This study presented an AI model to determine the risk of T2DM patients to develop to CHD, which has potential value in providing early warning personalized guidance of CHD risk for both T2DM patients and clinicians.

## Introduction

Diabetes mellitus (DM) is a serious and chronic disease resulted from the pancreatic beta-cells’ insulin secretion disorder^1–3^. In 1980, 108 million persons were diagnosed as diabetes while the number is increased to 463 million (4.2 million death) in 2019 all over the world, which was growing rapidly in the past decade according to the World Health Organization (WHO) and International Diabetes Federation (IDF)^1,4^. Currently, it has become one of the top 10 causes of death and IDF predicted that the number of DM patients will climb to over 700 million adults by 2045^4^. Moreover, DM can be briefly classified to type 1 (T1DM) and type 2 (T2DM), and the two types are totally different in clinical therapy^5^. Asia especially China could be considered one dominant area of T2DM due to a large amount of population base^6,7^. T2DM can result in a number of complications, such as macrovascular diseases, for example, cardiovascular disease (CVD), and microvascular diseases, for example, kidney, the retina and the nervous system diseases^7^. Even worse, T2DM may cause dementia and cognitive impairment, thereby reducing sensitivity to diabetes complications for T2DM patients^8^. It is known that the incidence of heart disease such as heart failure (HF), cardiac dysfunction in individuals with T2DM is much higher than those without T2DM^9^. Specifically, coronary heart disease (CHD) represents one of the most common and severe diabetes complications^10^.

CHD is a disease of the less blood supplying to heart muscle vessels^11^ manifested as hyperlipidaemia, myocardial infarction, and angina pectoris^12–14^ and ~ 17.7 million people perished from CHD in 2015^11,15^. Only in the United States, 18.2 million adults over 20 have CHD which take parts 6.7% of total population^16^ and CHD caused 363,452 death in 2017^17^. It is known that individuals’ basic information like gender and age, and blood test indexes such as blood pressure, total cholesterol (TC), low-density lipoprotein cholesterol (LDL-C), high-density lipoprotein cholesterol (HDL-C) as well as smoking behaviour, diabetes status can be considered as risk factors of causing CHD^18–20^. Therefore, the early diagnosis of CHD is important while it is not easy^21^. Given the high prevalence and mortality rate of CHD, it is thus important to predict CHD risk for individuals. For doing so, a number of models for predicting CHD have been proposed using mathematical models like cox regression^19,22,23^ and machine learning models like neural network^15,21,24^. These models were designed for the general population, however, a model specifically built for predicting CHD risk in T2DM patients is still not available. Moreover, 68% of the 65-year-old-or-older diabetes patients dead from some form of heart diseases like CHD^25^ and diabetes patients have 2 to 4 times higher risk of developing CHD than others^26^. Given the huge number of T2DM patients, it is thus quite important to evaluate the risk of developing CHD for T2DM patients.

In this study, we proposed an AI (random forest) based model to predict the risk of developing CHD for individuals with T2DM. As a result, the predictive model achieved an AUC of 0.77 (fivefold cross-validation) in the training dataset and an AUC of 0.80 in the testing dataset, respectively. Moreover, the model achieved an AUC of 0.71 on a totally independent dataset including 1,253 newly recruited T2DM patients. In addition, a risk contribution model was built to quantify the importance of each feature for a given T2DM individual. Finally, we implemented a web server for the predictive model.

## Methods

### Study subjects

In this study, all procedures complied with the Helsinki Declaration for investigation of human subjects. The study received ethical approval from the competent Institutional Review Boards of Lu He hospital. All subjects supplied written informed consent.

### Datasets

From January 2017 till June 2019, 1,357 subjects with T2DM were recruited in the study. Patients with T2DM were recruited from the Inpatient Department of Endocrinology in Lu He hospital. Exclusion criteria included any history or active treatment of cancer, pregnancy, cognitive inability as judged by the interviewer, any serious medical condition which would prevent long-term participation, the language barrier. Patients with other specific types of diabetes and patients with gestational diabetes mellitus were excluded. Finally, a total of 1,273 patients were enrolled in our study and all of them successfully underwent the medical history-taking included the history of smoking, alcohol, medical treatment, and history of CHD, hypertension and diabetes. All the features included are listed in Table 1. Among the 1,273 samples, 969 are diabetes patients without CHD (negative samples) and 304 are diabetes patients with CHD (positive samples). Next, we randomly selected 4/5 of positive samples and 4/5 of the negative samples as the training dataset. The rest samples are taken as the independent testing dataset. Finally, to confirm the accuracy of the presented predictive model, we recruited 1,253 totally new T2DM patients (200 positive samples and 1,053 negative samples) from the Outpatient Department of Endocrinology in Lu He hospital and determine the related information required by the model. Data is loaded and preprocessed by Pandas 0.25^27^ which is a package in Python 3.7.

**Table 1:** Brief description of each feature in the dataset with 1,273 subjects.

	Non-CHD	CHD
**Number of subjects (%)**	969 (76.1)	304 (23.9)
Female	468 (48.2)	161 (47.04)
Smokers	370 (38.11)	129 (42.43)
Drinking alcohol	309 (31.82)	97 (31.91)
**Mean (SD/95%CI) of characteristic**		
Age	54.07 ± 14.30	64.91 ± 9.75
Course of hypertension, y	5.45 (4.92, 5.98)	10.33 (9.10, 11.55)
Course of diabetes, y	7.26 (6.80, 7.73)	11.35 (10.38, 12.36)
Systolic pressure (mmHg)	129 ± 18.74	128.68 ± 20.94
Diastolic pressure (mmHg)	77.11 ± 11.61	72.80 ± 12.70
Heart rate (beats per minute)	83.42 ± 12.97	76.94 ± 11.13
Body mass index (kg/m^2^)	26.12 ± 4.04	26.38 ± 3.72
Waist hip rate (W/R)	0.94 ± 0.07	0.95 ± 0.07
**Biochemical data**		
Blood platelet (*10^9^/L)	225.38 ± 68.91	205.14 ± 82.72
Hemoglobin A1c (%)	9.84 ± 2.28	9.38 ± 2.20
Serum creatinine (µmol/L)	67.25 ± 27.66	76.21 ± 31.97
Uric acid (mmol/L)	319.74 (312.95, 326.53)	334.91 (321.41, 346.41)
Serum triglyceride (mmol/L)	2.03 (1.88, 2.17)	1.77 (1.58, 1.95)
Total cholesterol (mmol/L)	4.71 ± 1.21	4.13 ± 1.17
LDL cholesterol (mmol/L)	3.02 ± 0.88	2.57 ± 0.87
HDL cholesterol (mmol/L)	1.08 ± 0.27	1.04 ± 0.28
Fasting blood glucose (mmol/L)	9.07 (8.78, 9.38)	8.61 (8.15, 9.08)
**Indexes of insulin secretion**		
Insulin 0 h (unit/mL)	17.92 (14.26, 21.60)	29.63 (18.98, 40.30)
Insulin 1 h (unit/mL)	58.56 (53.64, 64.38)	78.72 (64.38, 93.06)
Insulin 2 h (unit/mL)	65.09 (59.82, 70.36)	84.83 (69.47, 100.19)
Insulin 3 h (unit/mL)	53.35 (48.16, 58.52)	75.46 (60.06, 90.87)
c-peptide 0 h (ng/mL)	1.57 (1.51, 1.64)	1.82 (1.68, 1.95)
c-peptide 1 h (ng/mL)	2.75 (2.63, 2.88)	2.82 (2.62, 3.01)
c-peptide 2 h (ng/mL)	4.13 (3.94, 4.33)	4.16 (3.84, 4.48)
c-peptide 3 h (ng/mL)	4.24 (4.05, 4.44)	4.50 (4.14, 4.86)

### Diagnostic criteria

Diagnostic criteria of T2DM are in line with guidelines for the prevention and treatment of type 2 diabetes in China (2017 edition). Diagnostic criteria of CHD meet guidelines for the diagnosis and treatment of stable coronary heart disease (SCAD) (2018 edition). Hypertension was a blood pressure of at least 140 mmHg systolic or 90 mmHg diastolic or use of antihypertensive drugs.

### Biochemical measurement

All participants suffered overnight fasting before venous blood samples were drawn. We aim to measure the total and differential white blood cell count, red blood cells, platelets, hemoglobin A_1c_ (HbA_1c_), serum creatinine (SCr), uric acid (UA), serum triglyceride (TG), TC, LDL-C, HDL-C, fasting blood glucose (FBG), D-dimer, C-reactive protein (CRP), gamma-glutamyl transpeptidase (GGT). We also collected insulin and C-peptide levels of 0, 1, 2, 3 h when patients went through Oral Glucose Tolerance Test (OGTT). All indexes were measured in the central laboratory in Lu He hospital.

### Information entropy function-based feature selection method

Information entropy is a conventional concept in information theory which is proposed by C. E. Shannon in 1948 and it can quantitatively describe the information contained in a series of data. Here, we use the information entropy function and Gini impurity function to check the information hidden in each feature. One feature will get a higher score if this series of data contain more information for classification and vice versa. The information entropy function-based feature selection method is implemented by using random forest model with 500 decision trees in sci-kit-learn 0.22^28^ in Python 3.7. Because the decision tree classifier makes decisions based on the entropy function, the average importance of each feature in 500 decision trees is calculated.

### Random forest based predictive model

Random forest (RF)^29^ is a conventional ensemble model for machine learning. It uses the information entropy function or Gini impurity function for discrimination. Here, we proposed a RF-based predictive model (DCHD, Diabetic Coronary Heart Disease) with Gini impurity as an entropy function, which is also known as Classification And Regression Tree (CART)^30^. The Gini impurity function of a decision tree node with dataset $D$ is defined as

$$Gini\left( D \right) = 1 - \sum\limits_{i = 1}^C {p_i^2}$$

where ${p}_{i}$ is the probability of belonging to class $i$ in dataset $D$ and $i=\mathrm{1,2},...,C$. The dataset $D$ will be divided into 2 subsets on this tree node based on the criterion $A=a$ which is the minimal Gini gain point defined as

$$Gain(D,a)=\frac{|{D}_{1}|}{|D|}Gini({D}_{1})+\frac{|{D}_{2}|}{|D|}Gini({D}_{2})$$

where ${D}_{i}$ is the subsets after applying division $A=a$ (${D}_{1}=\left\{d\in D|d\le a\right\},{D}_{2}=\{d\in D|d>a\}$). The number of trees is set to 500 and no tree depth limitation is applied to get a more precise and robust model. This model is implemented by using sci-kit-learn 0.22^28^ in Python 3.7.

### Risk contribution model

We also performed an analysis of how much contribution a feature has by using the proportion method to calculate the contribution of each feature for individuals which is described in the following equations.

$${F}_{i}^{k}=\left[{f}_{0}^{k}, {f}_{1}^{k}, \dots ,{f}_{i-1}^{k}, 0, {f}_{i+1}^{k}, \dots , {f}_{m-1}^{k}\right]$$

$${S}_{i}=RF\left({F}^{k}\right)-RF\left({F}_{i}^{k}\right)$$

where ${f}_{i}^{k}$ is the value of the ${i}$th feature for the ${k}$th sample and $m$ is the total number of selected features. So, ${F}_{i}^{k}$ represents the feature vector where the ${i}$th feature value is zero and ${F}^{k}$ is the original feature vector; $RF$ represents the probability of developing CHD in the predictive model. The zero value in the latent represents the mean value of that feature because data is standardized.

Note that one contribution can not only be positive but also negative because some of the features can be normal and make the risk probability drop a little bit.

### Web server for the predictive model

In order to make the risk prediction model available for all T2DM patients, we built a web server (https://www.cuilab.cn/dchd). The server-side was performed by Django 2.2.5 package in Python 3.7 and the user interface was built using Bootstrap 4 and HTML 5.

## Results

### Risk features selection and validation

**Figure 1:**
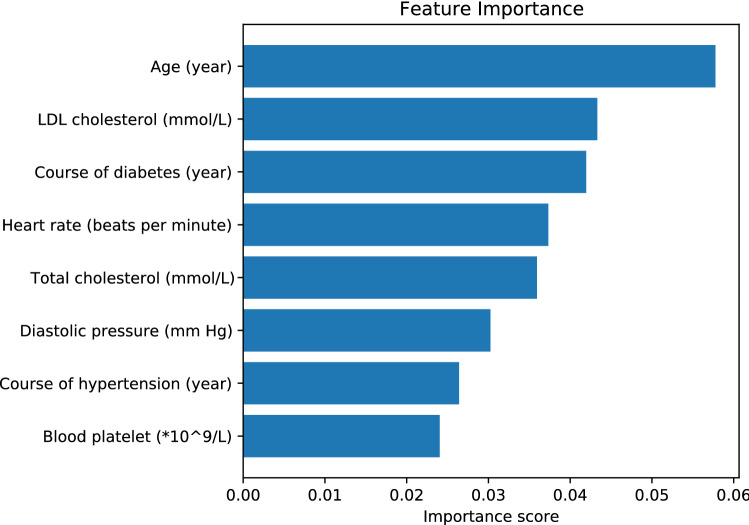
The importance scores of the top 8 features calculated by information entropy function-based feature selection model.

**Figure 2:**
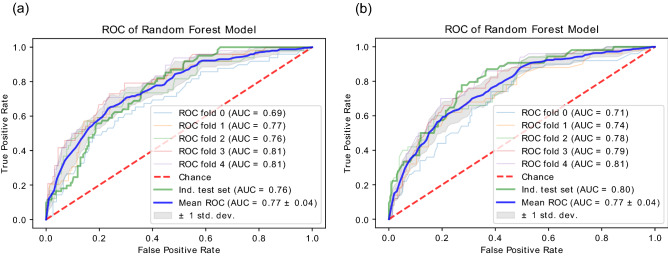
The ROC and the AUC of the predictive model on both the fivefold cross-validation (blue) and the independent testing dataset (green) using the dataset with all features (**a**) and using the dataset with top 8 selected features (**b**).

Fewer features normally make it more convenient for T2DM patients and clinicians to use the model and make the model more robust while it could decrease the performance of the model. To find a balance between the prediction robustness, convenience, and accuracy, here information entropy function-based feature selection method is applied to the whole dataset with the total 52 features. All these features are treated as input features of a random forest model and train the model with 500 decision trees. From this model, the entropy function represents the importance of each feature. And we found that the information entropy function and Gini impurity function make little difference in feature selection. Here we choose Gini impurity as the criteria function. The higher scores the feature got, the more information the feature contains. Next, the top 8 features (Age, LDL-C, Course of diabetes, TC, Heart rate, Diastolic pressure, Blood platelet, Course of hypertension) are selected and they contribute 30% among all the features and each of the rest features can do just little contribution (less than 2.3%) on distinguishing CHD from non-CHD in T2DM patients. The information contributions of the selected features are sorted and shown in Fig. 1. A more general and robust model is built using these selected features with almost the same performance compared with the original model. (Fig. 2a,b).

### Performance of the predictive model

A number of metrics are often used to evaluate the prediction performance of machine learning models, such as true positives (TP), true negatives (TN), false positives (FP) and false negatives (FN). Here, TP and TN are the correctly classified CHD and non-CHD, respectively; FN represents CHD that are misclassified as non-CHD; non-CHD incorrectly classified as CHD is defined as FP. And then several standard performance metrics are applied to describe the model performance based the metrics including accuracy (ACC), true positive rate (TPR) also known as recall rate, false positive rate (FPR), precision rate and F1 score.

$$ACC=\frac{\left(TP+TN\right)}{\left(TP+TN+FP+FN\right)}$$

$$TPR=\frac{TP}{\left(TP+FN\right)}=Recall$$

$$FPR=\frac{FP}{\left(TN+FP\right)}$$

$$Precision=\frac{TP}{TP+FP}$$

$$F1=\frac{2*Precision*Recall}{Precision+Recall}$$

As a result, the presented model achieved an AUC of 0.77 for fivefold cross-validation and an AUC of 0.80 in the independent testing dataset (Fig. 2b). And the performance scores introduced before are listed in Table 2.

**Table 2:** Performance scores of the predictive model.

	ACC	TPR (recall)	FPR	Precision	F1
Training set	0.7892	0.2041	0.0129	0.8	0.2712
	0.8088	0.5306	0.0968	0.625	0.5618
	0.701	0.8367	0.3419	0.4333	0.5612
Testing set	0.7922	0.2131	0.0155	0.7857	0.2933
	0.7255	0.5082	0.2113	0.4348	0.4615
	0.6353	0.8033	0.4175	0.375	0.5079

### Performance of the predictive model on a newly recruited independent testing dataset

**Figure 3:**
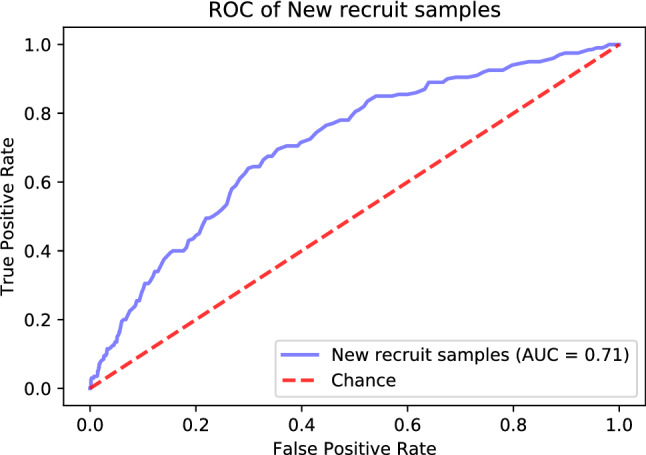
The ROC and AUC of the predictive model on the newly recruited dataset.

To further confirm the robustness and performance of our model, we newly recruited 1767 patients with T2DM from the Outpatient Department of Endocrinology. Among these patients, 1,253 subjects were finally enrolled including 200 with CHD and 1,053 without CHD. As a result, our model achieved an AUC of 0.71 in the newly recruited independent testing dataset (Fig. 3). In addition, the performance scores for this dataset are listed in Table 3.

**Table 3:** Performance scores of the predictive model on the newly recruited dataset.

	ACC	TPR (recall)	FPR	Precision	F1
New set	0.834	0.2	0.0437	0.4524	0.2676
	0.7558	0.5	0.1994	0.3257	0.3929
	0.5499	0.815	0.5071	0.2331	0.3605

### Risk contributions of features

**Figure 4:**
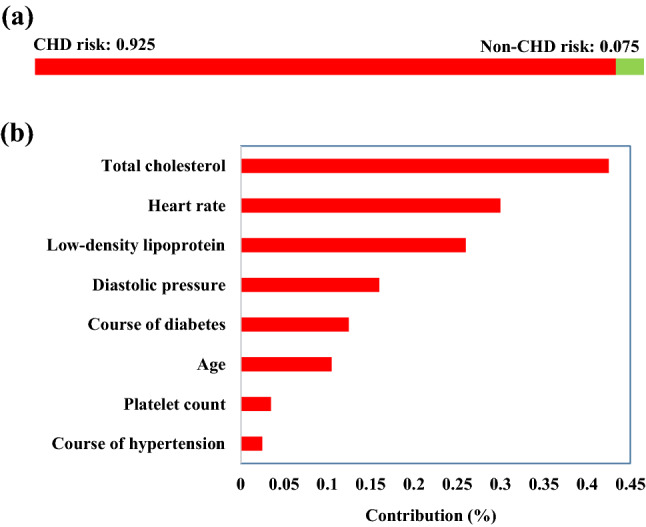
The predicted result of a case study. (**a**) The predicted CHD risk (red bar) or non-CHD risk (green bar). (**b**) The predicted feature contributions for the input individual.

Risk contribution of a feature represents an indication of how much this feature impacts the risk of developing CHD. As a case study, here is a T2DM patient whose data for each feature is as follows, Age: 68, Low-density lipoprotein: 1.92, Course of diabetes: 20, Total cholesterol: 3.32, Heart rate: 65, Diastolic pressure: 67, Platelet count: 340, Course of hypertension: 3. This individual was predicted to be at a high risk to develop to CHD (0.925) using our model (Fig. 4a). And by risk contribution model, the calculated scores of risk factors are as follows, Age: 0.105, Low-density lipoprotein: 0.26, Course of diabetes: 0.125, Total cholesterol: 0.425, Heart rate: 0.3, Diastolic pressure: 0.16, Platelet count: 0.035, Course of hypertension: 0.025 (Fig. 4b). All the result was shown on the web server. In the first bar plot, the length of the red bar represents the probability of developing to CHD and the length of the green bar represents the probability of non-CHD. Moreover, the risk factors’ contributions are sorted and plotted in the bottom figure. The contributions thus can provide advice on daily diet and clinical treatment for individuals.

### Web server

The home page of the webserver is shown as Figure S1. For single instance prediction (Figure S1a), users can input the value of the features needed by the model and click the “Run” button. Then, the model will analyze the input data and output the result in a new page (the result figures like Fig. 4 will be shown). Clicking the “Example” button, the data for a case study will be entered automatically. For multiple instances prediction (Figure S1b), users can paste a CSV format text and click “Run” to get a batch of prediction results.

## Discussion

T2DM is a common disease and often resulted in death due to severe complications, among which, CHD is one most common and severe one. Given the huge number of T2DM patients, it thus becomes increasingly critical to quantitatively evaluate the risk for a T2DM patient to develop to CHD in the near future.

In this paper, we presented an AI-based model to predict the risk of developing CHD for T2DM patients. We first proposed a feature selection model to confirm risk factors. Then, a predictive model was built to predict CHD among T2DM patients. As a result, the model achieved an AUC of 0.77 on fivefold cross-validation and 0.80 on an independent testing dataset. Moreover, the presented model achieved an AUC of 0.71 on a newly recruited dataset. Finally, a risk proportion model was built for individuals to analyze the contribution of each feature to CHD risk.

The predictive model is available online for users to do a self-checking and it can be treated as the early-warning of developing CHD if the probability is high enough. Moreover, clinicians can use the online web server as an auxiliary tool to determine potential CHD risk for a T2DM patient. The risk contribution results can also be used by doctors to design personalized treatment strategies for different patients.

Furthermore, the blood test can be considered as a very basic and cheap test in physical checkup and the features used in the predictive model are mostly from the blood test. That is, the risk prediction model is convenient to use for self-checkup and more detailed physical checkup needs to be done if a high risk is reported by the model.

The current model can be improved in the following aspects. Firstly, the used features in this study are limited. Therefore, the model could be improved if more valuable features from other aspects (such as medication history and heart imaging data) are included in the future. Secondly, there may be non-linear compositions of known features (such as age * blood pressure). Although the random forest model is not a linear model, it cannot detect and explain all kinds of non-linear compositions. So, the error accumulates when the number of non-linear compositions increases in real situations. Therefore, the non-linear analysis would be of help in improving this model in the future. Besides, one more important limitation is that both the training dataset and independent validation dataset are from the same hospital and there is no tracking data of the patients with the non-CHD clinical diagnosis but high risks from the prediction model, which may be a source of bias. Hence, the model would be more robust and convincible if the training data are from multi-source and cohort tracking data is included.

In summary, we presented a reliable AI model to predict CHD risk for T2DM patients, which could be of help for precision DM care. Finally, we will continuously update the predictive model to achieve better performance and to provide greater help for the precision medicine of T2DM patients.

## Supplementary information

Supplementary Figure Legends.

Supplementary Figure S1.
