# Supplementary material for: AI-based prediction for the risk of coronary heart disease among patients with type 2 diabetes mellitus
Source: Sci Rep. 2020 Sep 2;10:14457. doi: 10.1038/s41598-020-71321-2 (PMC7467935; doi:10.1038/s41598-020-71321-2)
Supplement: Supplementary file 1 — Supplementary Figure Legends. [file 41598_2020_71321_MOESM1_ESM.docx]

**Figure S1. The home page of the web server of the predictive model.** (a) Single instance prediction. (b) Multiple instance prediction.
