# Supplementary material for: AI-based prediction for the risk of coronary heart disease among patients with type 2 diabetes mellitus
Source: Sci Rep. 2020 Sep 2;10:14457. doi: 10.1038/s41598-020-71321-2 (PMC7467935; doi:10.1038/s41598-020-71321-2)
Supplement: Supplementary file 2 — Supplementary Figure S1. [file 41598_2020_71321_MOESM2_ESM.pdf]

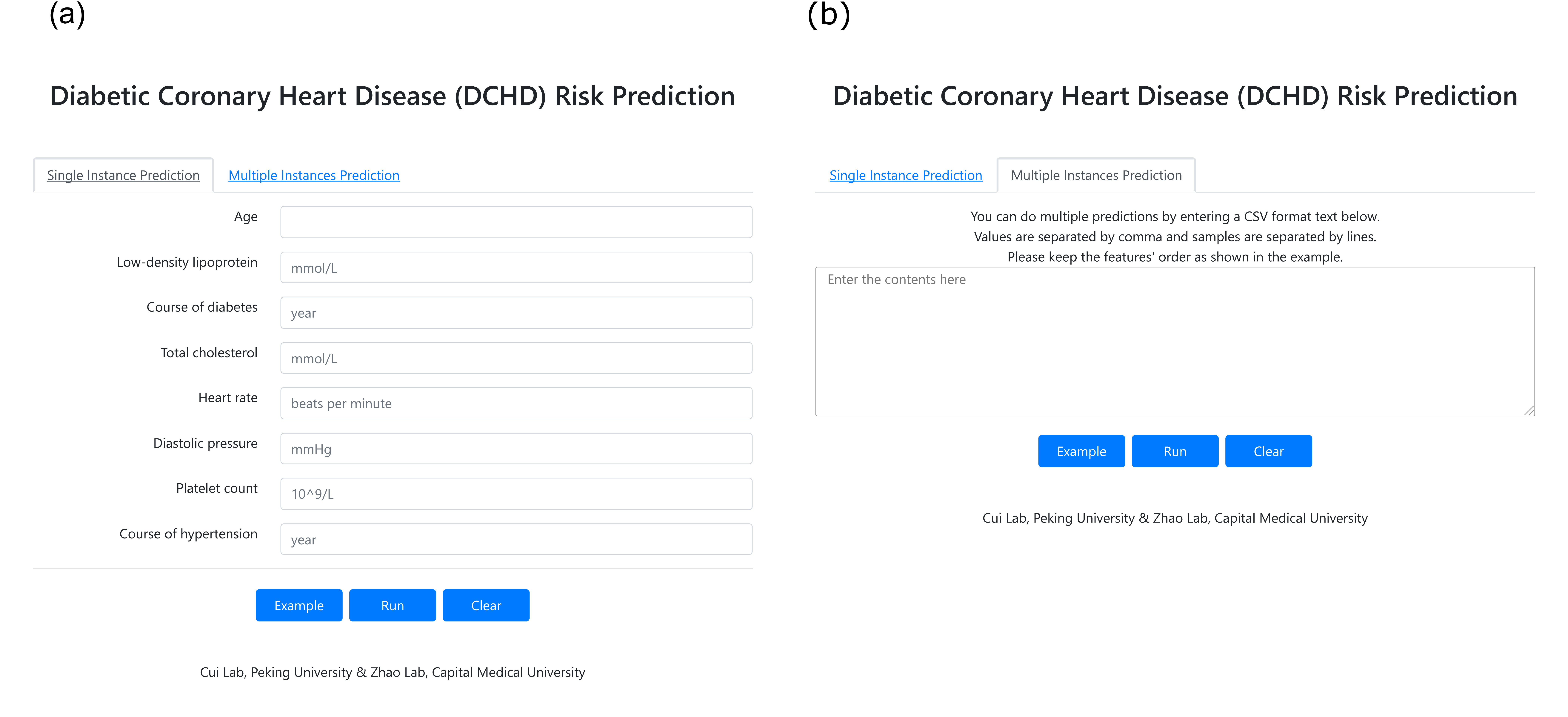
(a)
Diabetic Coronary Heart Disease (DCHD) Risk Prediction
Single Instance Prediction
Multiple Instances Prediction
Age
Low-density lipoprotein
mmol/L
Course of diabetes
year
Total cholesterol
mmol/L
Heart rate
beats per minute
Diastolic pressure
mmHg
Platelet count
10^9/L
Course of hypertension
year
Example
Run
Clear
Cui Lab, Peking University & Zhao Lab, Capital Medical University
(b)
Diabetic Coronary Heart Disease (DCHD) Risk Prediction
Single Instance Prediction
Multiple Instances Prediction
You can do multiple predictions by entering a CSV format text below.
Values are separated by comma and samples are separated by lines.
Please keep the features' order as shown in the example.
Enter the contents here
Example
Run
Clear
Cui Lab, Peking University & Zhao Lab, Capital Medical University
